# Alternative and Adjunct Treatments for Scoliosis: A Google Trends Analysis of Public Popularity Compared With Scientific Literature

**DOI:** 10.7759/cureus.38682

**Published:** 2023-05-07

**Authors:** Logan Laubach, Benjamin Chiang, Viraj Sharma, Jonathon Jacobs, John W Krumme, Victoria Kuester

**Affiliations:** 1 Orthopaedic Surgery, Virginia Commonwealth University School of Medicine, Richmond, USA; 2 General Surgery, Riverside University Health System Medical Center, Riverside, USA; 3 Biostatistics, Virginia Commonwealth University School of Medicine, Richmond, USA; 4 Orthopaedic Surgery, University of Missouri Kansas City School of Medicine, Leawood, USA

**Keywords:** evidence based medicine, alternative adjunct treatments, public interest, scoliosis, google trends

## Abstract

Purpose

As Google searches have often been found to provide inaccurate information regarding various treatments for orthopedic conditions, it becomes important to analyze search trends to understand what treatments are most popularly considered and the quality of information available. We sought to compare the public interest in popular adjunct/alternative scoliosis treatments to the published literature on these topics and assess any temporal trends in the public interest in these treatments.

Methods

The study authors compiled the most common adjunct/alternative treatments for scoliosis on PubMed. Chiropractic manipulation, Schroth exercises, physical therapy, pilates, and yoga, along with "scoliosis," were each entered into Google Trends, collected from 2004 to 2021. A linear regression analysis of covariance (ANCOVA) was done to determine whether there was a linear relationship between Google Trends' popularity and PubMed publication data. The seasonal popularity of the terms was assessed using locally estimated scatterplot smoothing (LOESS) regression.

Results

Google Trends and publication frequency linear regression curves were different for chiropractic manipulation (p < 0.001), Schroth exercises (p < 0.001), physical therapy (p < 0.001), and pilates (p = 0.003). Chiropractic manipulation (p < 0.001), Schroth exercises (p = 0.003), and physical therapy (p < 0.001) had positive trends, and yoga (p < 0.001) had a negative trend. Chiropractic manipulation and yoga were more popular in the summer and winter months.

Conclusion

Google Trends can provide orthopedic surgeons and other healthcare professionals with valuable information on which treatments are gaining popularity with the public, so physicians may specifically inform themselves prior to patient encounters, leading to more productive shared decision-making.

## Introduction

The Internet has become a primary tool for searching for common orthopedic conditions since 1999 [1,2]. Previous studies have demonstrated that 97% of parents used the Internet for their children’s orthopedic conditions prior to their first appointment, with 6% of these searches pertaining to scoliosis [3]. Additionally, it was found that 90% of patients believed their internet sources to be reliable sources of information. However, one study that reviewed top Internet sources for pediatric orthopedic conditions determined that only about 15% of sources had high-quality information [4].

Google is becoming increasingly popular for health inquiries, and Google Trends can be used to track the popularity of these inquiries over time. Studies involving Google Trends have been utilized to examine the popularity of various alternative treatments for common orthopedic conditions [5,6]. There have been discrepancies between the information on various treatments for orthopedic conditions found on a quick Google search and their validity and safety [4]. Understanding which orthopedic topics are rising in popularity is vital to providing patients with accurate and safe information.

Given that scoliosis screening is prevalent at schools and well-child exams throughout the United States, with a prevalence of 0.47-5.2%, providing parents with accurate information regarding common scoliosis questions is important [7]. We sought to compare the public interest in popular adjunct/alternative scoliosis treatments via Google Trends to the published medical literature on these topics and assess if there were any temporal trends in the public interest in these treatments.

## Materials and methods

Search terms

Three study authors, including the senior orthopedic surgeon author, compiled the top five most common adjunct/alternative treatments for scoliosis based on a literature search on PubMed ((scoliosis[Title/Abstract]) AND ((alternative[Title/Abstract]) OR (adjunct[Title/Abstract])) AND (treatment[Title/Abstract])). The top five most popular alternative/adjunct treatment terms were "Chiropractic Manipulation," "Schroth exercises," "physical therapy," "pilates," and "yoga." These terms were combined with "scoliosis" and were each entered into Google Trends, an online tool that shows the popularity of searchable terms on the search engine Google [8,9].

Google Trends data collection

Google Trend data are normalized as a ratio of the relative number of searches for each search term over the total number of searches within that geographical region and time frame that the search was performed in [8]. These data are normalized on a scale of 0 to 100 (a higher number indicating higher popularity) as a ratio of the relative number of searches for each search term over the total number of searches over time.

Google Trend aggregate search term data were compiled for each alternative treatment search term over the last 17 years, from January 1, 2004, to January 1, 2021, from searches performed in the United States. The start date was chosen because 2004 was the earliest date with Google Trends data. 2022 data were not complete at the time of this study and were thus excluded from the analysis. In addition, the same terms "Chiropractic Manipulation," "Schroth exercises," "physical therapy," "pilates," and "yoga" combined with "scoliosis" were also entered into PubMed to trend the number of publications from 2004 to 2021 to mirror the Google Trends data timeline. These search term combinations were queried using the advanced search builder feature in PubMed, searching the "title/abstract" field. Article titles and abstracts were then reviewed for relevance for articles not specifically addressing scoliosis, and each associated alternative/adjunct treatment was removed from the analysis.

Statistical analysis

The biostatistics laboratory at our institution was consulted for statistical analysis. Linear regression analysis was done using SPSS (Version 28.0.1.0, IBM Corp., Armonk, NY) to determine whether there was a linear relationship between time and Google Trends popularity as well as time and PubMed publication data, using p<0.05 to determine significance. JMP Pro (Version 16.1.0, SAS Institute, Inc., Cary, NC) was used to create the linear regression graphs and compare the linear regression analysis of covariance (ANCOVA) between Google Trends and PubMed publication results for each alternative/adjunct treatment, using p<0.05 to determine the significance. Linear regression lines for Google Trends popularity and PubMed publications were normalized to the largest value, respectively, and graphed using GraphPad Prism (version 9.4.1 for Windows, GraphPad Software, Inc., La Jolla, CA).

Mann-Kendall trend tests were performed on all five terms of the Google Trends time series to determine if there were significant overall trends in the data presented. A non-parametric regression technique called locally estimated scatterplot smoothing (LOESS) regression was applied to each of the five search terms in the Google Trends time series to determine if the terms were more popular during specific seasons. For those time series with noticeable seasonal components, summer (April-October) and winter (November-March) medians were computed, and two-way Wilcoxon signed-rank tests were performed to see if these medians differed from each other. These analyses were performed in R (version 4.2.2, RStudio, Boston, MA) with the use of the forecast package, and a nominal α = 0.05 type-I error rate was used.

Institutional Review Board (IRB) approval was not necessary since these data were publicly available.

## Results

**Figure 1:**
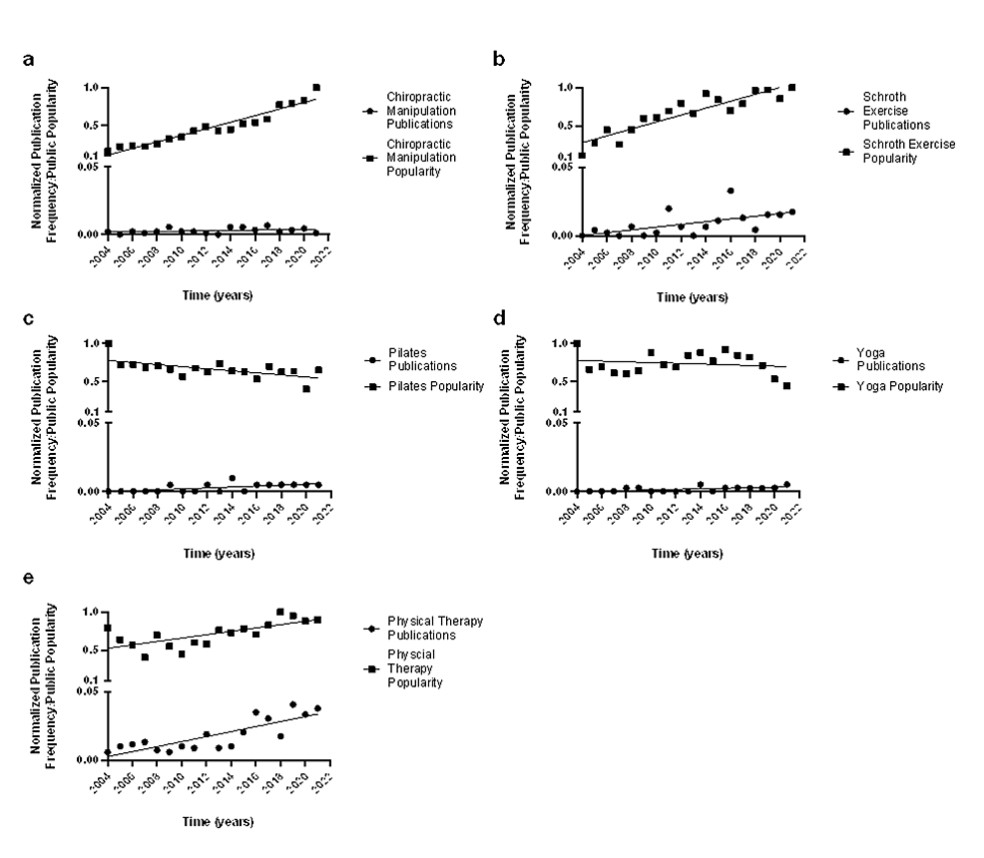
Linear regression analysis of (a) chiropractic manipulation, (b) Schroth exercises, (c) pilates, (d) yoga, (e) physical therapy PubMed publication frequency vs Google Trends popularity from 2004 to 2021

For alternative/adjunct treatments for scoliosis on Google Trends, chiropractic manipulation (R2 = 0.9, p < 0.001), Schroth exercises (R2 = 0.8, p < 0.001), physical therapy (R2 = 0.5, p = 0.001), and pilates (R2 = 0.4, p = 0.006) demonstrated significant linear relationships with time. Yoga (p = 0.5) did not have a significant relationship with time. For publications, physical therapy (R2 = 0.7, p < 0.001), pilates (R2 = 0.4, p = 0.005), yoga (R2 = 0.4, p = 0.006), and Schroth exercises (R2 = 0.4, p = 0.008) demonstrated significant linear relationships with time, while chiropractic manipulation (p = 0.161) did not (Table 1 and Figure 1).

**Table 1:** Linear regression analysis of Google Trends and PubMed publication frequency (2004-2021) *Statistically significant at p < 0.05, demonstrating a significant linear relationship from 2004 to 2021 ^a^Adapted from Hinkle DE, Wiersma W, Jurs SG. Applied Statistics for the Behavioral Sciences. 5th ed. Boston: Houghton Mifflin: 2003.

Alternative/adjunct treatment	R^2^ for Google Trends popularity	p for Google Trends popularity	Strength of correlation*	R^2^ for publication frequency	p for publication frequency	Strength of correlation^a^	p for Google Trends popularity vs publication frequency
Chiropractic manipulation	0.9	<0.001*	Very high	Not applicable	0.161	Not applicable, no linear relationship	<0.001*
Schroth exercises	0.8	<0.001*	Very high	0.4	0.008*	Moderate	<0.001*
Physical therapy	0.5	0.001*	Moderate	0.7	0.001*	High	<0.001*
Pilates	0.4	0.006*	Low	0.4	0.005*	Moderate	0.003*
Yoga	Not applicable	0.483	Not applicable, no linear relationship	0.389	0.006*	Moderate	0.460

The ANCOVA between Google Trends and publication frequency linear regression curves were statistically significant for chiropractic manipulation (p < 0.001), Schroth exercises (p < 0.001), physical therapy (p < 0.001), and pilates (p = 0.003), while yoga (p = 0.460) did not reach statistical significance.

Within the Google Trends data, the terms chiropractic manipulation (p<0.001), Schroth exercises (p=0.003), and physical therapy (p<0.001) had significant positive trends, and yoga (p<0.001) had a significant negative trend, while pilates did not reach a significant trend (Table 2). Chiropractic manipulation and yoga were more popular in the summer and winter months, although there was no difference between the seasons within each search term.

**Table 2:** Time series analysis of google trends search term popularity

Search terms	Mann-Kendall trend test	Seasonality present?	Summer median popularity	Winter median popularity	Wilcoxon signed rank test
Chiropractic manipulation	p < 0.001, tau = 0.5	Yes	19	19	p = 0.896
Schroth exercises	p = 0.003, tau = 0.2	No	7.5	8	
Physical therapy	p < 0.001, tau = 0.2	No	8	6	
Yoga	p < 0.0001, tau = -0.3	Yes	19.5	18	p = 0.364
Pilates	p = 0.219, tau = −0.1	No	3	2	

## Discussion

While the Internet has become commonplace for looking up health information, the first results on Google may not be the most accurate [4]. Many health-related search queries yield advertisements and information possibly biased by financial gain [10]. Google Trends has become a popular and powerful research tool to assess public interest in various topics [11]. For example, Google Trends has been shown to predict local COVID-19 outbreaks by searching terms related to symptoms and vaccine information [12,13]. In the orthopedics realm, Google Trends has been used in a similar manner: tracking healthcare use for hand osteoarthritis and public interest in the risks and benefits of total hip and knee replacements [14-16]. Therefore, this powerful public dataset has validated itself as a way to inform physicians about popular public interest in various healthcare topics, even orthopedics.

The growth of published information (PubMed) and Internet information (as measured by Google Trends) between 2004 and 2021 for alternative and adjunct treatments for scoliosis is different (Table 1). Certain treatments such as chiropractic manipulation, physical therapy, and Schroth exercises have risen in popularity, while yoga has declined in interest. Google Trends can assess these changes in the public's popularity of alternative/adjunct treatments for scoliosis. These differences in Google Trends have been utilized to gauge public interest in platelet-rich plasma as an alternative treatment for hip and knee arthritis. Interest has risen significantly since 2009, despite varying evidence of its efficacy as well as possible complications related to this invasive treatment [15]. Similar research has looked into the rising interest in using stem cell therapy as well as CBD for the treatment of arthritis, despite conflicting evidence regarding efficacy and safety profiles [6]. These differences have been found in popular alternative/adjunct treatments for common orthopedic conditions and align with our findings with scoliosis (Table 1).

For example, the term chiropractic manipulation with scoliosis has had a significant linear growth in terms of Internet popularity, while publication information does not share the same trend (Figure 1 and Table 1). The current literature on chiropractic manipulation for patients with scoliosis is limited to case reports and small observational studies with no controls and some reported serious adverse effects [17-19]. Schroth exercises also differed in the strength of the correlation between Google Trends and PubMed information. However, the published literature on Schroth exercises is greatly in favor of its use both as an adjunct to standard of care (bracing with Cobb angles <45°) and as an alternative treatment to bracing in patients with less severe Cobb angles [20-22]. Physical therapy, yoga, and pilates have shown similar beneficial outcomes as adjunct treatments for scoliosis, although the literature on the latter two is less robust [23-25]. The Scoliosis Research Society provides some information on physical therapy and some additional alternative/adjunct treatments for scoliosis for parents and patients, which may contribute to the public interest in these [26]. These data highlight the importance of the differences in public interest compared to both the quantity and quality of published literature on these alternative/adjunct treatments for scoliosis. Google Trends can provide insight to orthopedic surgeons as to which specific treatments are gaining popularity and guide their literature search to provide the most accurate and up-to-date information to their patients.

Google Trends has been used as a powerful tool in the healthcare realm since it has been able to predict popular public trends over time, from the COVID-19 outbreak to specific cancer treatments [11,13]. This may inform physicians about the types of questions that their patients may have as they come across more information online. In this study, chiropractic manipulation, Schroth exercises, and physical therapy had significant increases in public interest, while yoga had a significant decrease. Additionally, chiropractic manipulation and yoga were more popular during both the summer and winter seasons (Table 2). Spinal fusion surgeries tend to be more popular during the summer months as well as in December [27]. The increase in scoliosis surgeries around the summer and winter months would align with an increase in interest in alternative/adjunct treatments online. Public interest in foot and ankle pain treatment has been shown to have a seasonal component using Google Trends, with the most interest peaking during the summer months. This has been informative for orthopedic surgeons to predict relative emergency room and office visit volumes regarding foot and ankle trauma [28]. Determining both the general trends of public interest as well as the specific times of the year when these scoliosis treatments peak can guide orthopedic surgeons to narrow down their literature search prior to patient encounters for more productive and tailored treatment discussions.

Limitations of this study include a lack of data on Google Trends to differentiate between different types of scoliosis, such as idiopathic and neuromuscular scoliosis. Unfortunately, it was impossible to determine if patients searching for internet terms required correctional surgery or to characterize the extent of the scoliosis curve. This resulted in a dataset that was generalized to all forms and severities of scoliosis, providing a top-down view of the popularity of these alternative/adjunct treatments. We believe the data in this study to still be valuable as pediatric orthopedic surgeons treat a wide variety of scoliosis conditions and thus will come across patient questions across the specific subtypes of scoliosis. In addition, Google Trends is a measurement of public use of their parent search engine, Google, and thus may exclude those that use other popular search engines. However, Google is used more than 85% time as a search engine compared to its next closest competitor at just over 8%, so we believe any sampling bias to be minimal for results from other search engines [29]. Additionally, some research may not be PubMed-indexed and therefore could have been excluded from our search. The methods by which various search engines determine results are ambiguous. Search engines such as Google use proprietary algorithms to rank and present vast quantities of information with a focus on content, keywords, the "freshness" of how recently it was updated, and user engagement. More heavily searched websites and pages with both greater and more recent content will rank higher on the search result page and thus translate to Google Trends popularity [10,30]. Additionally, this study was limited to Google Trends data from the United States, while the PubMed publication data represent international scoliosis research. Therefore, there may be some incongruity in our results when comparing popular searches in the United States and international interest in scoliosis research. A more global study, including the comparison of different geographical regions, would provide insight into potential differences between countries and their scientific publications.

## Conclusions

These data highlight the differences in public interest in treatments for scoliosis that may or may not have peer-reviewed scientific information to justify their popularity and implementation. This trend, even for one pediatric orthopedic condition, becomes important, especially with studies showing that as little as 25% of current orthopedic information online is accurate. Google Trends information may further provide orthopedic surgeons with insight into alternative and adjunct treatments for conditions such as scoliosis and may inform future research into whether these treatments are viable and safe. Additionally, this tool can provide orthopedic surgeons with valuable information on which treatments are gaining popularity with the public, so they may specifically inform themselves prior to patient encounters, leading to more productive and shared decision-making.
